# Global research landscape and burden of disease in pediatrics: Identifying the research gaps to set directions for a new general pediatric journal

**DOI:** 10.1002/pdi3.2

**Published:** 2023-06-10

**Authors:** Janne Estill, Yaolong Chen

**Affiliations:** ^1^ Institute of Global Health University of Geneva Geneva Switzerland; ^2^ School of Basic Medical Sciences Lanzhou University Lanzhou China; ^3^ Chevidence Lab of Child and Adolescent Health Children's Hospital of Chongqing Medical University Chongqing China

**Keywords:** academic journals, bibliometric study, disease burden, pediatrics

## Abstract

Pediatrics intersects with almost the entire field of medicine, and thus the diversity of suitable publication platforms is particularly broad. We reviewed the current health issues among children and adolescents, the topics and scope of existing pediatric journals, as well as the distribution of a random selection of articles according to different health topics and journal types. Neurological disorders, mental health, and neonatal conditions were major contributors not only to loss of health and life among children and adolescents but also common themes of research articles and pediatric subspecialty journals. On the other hand, in some clinical areas, such as dermatology, the research does not necessarily meet the needs of practice. Methodological studies in pediatrics were rarely published, but they were common among preprints. We call for Pediatric Discovery to promote a research viewpoint that focuses on the child instead of individual conditions, pay particular attention to health concerns associated with the greatest burden and interactions between them, and promote the further standardization of reporting and methodologies that account for the unique characteristics of children and adolescents.

## Graphical abstract

Pediatrics is an extremely heterogeneous field of medicine. We reviewed the themes of existing pediatric journals and trends in recently published articles in pediatrics and found that although fields such as neurology are well covered by research findings, methodological research and some fields remain understudied. We call Pediatric Discovery to pay particular attention to the unique characteristics of children and the need for standardized reporting and tailored methodologies.

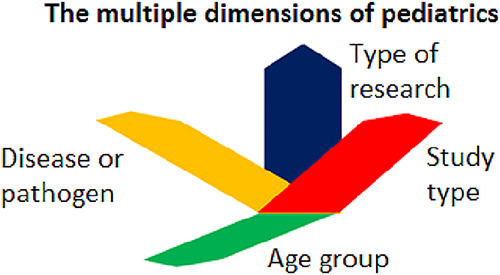

## 1 INTRODUCTION

**FIGURE 1:**
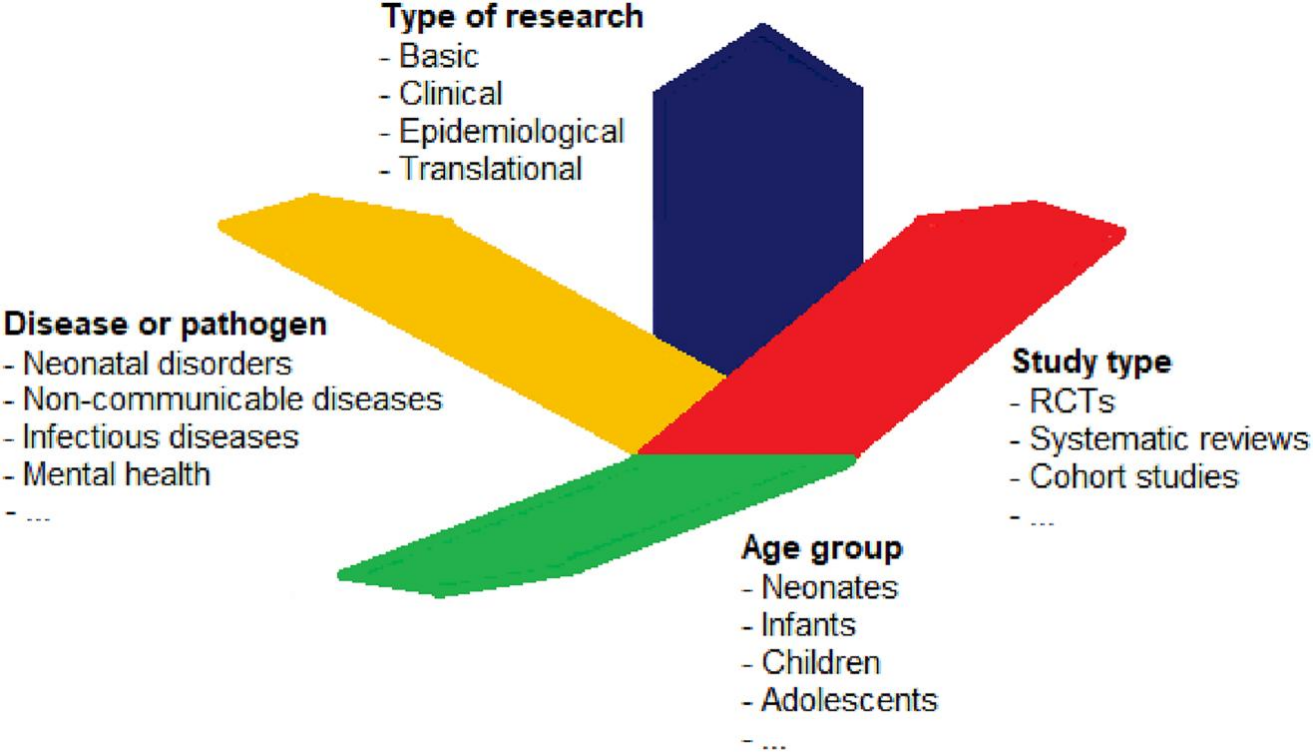
Dimensions of pediatric research.

There exists broad consensus that children are a priority population when health resources are limited.^1^ In a similar way, there is every reason to argue that pediatrics should also be a priority research area. Pediatrics is, however, a very special and unique field of medicine. It expands in multiple dimensions (Figure 1), crossing over the entire spectrum of medicine: from chronic diseases to acute infectious disease epidemics, from common childhood diseases to life‐threatening rare conditions, and from public health and prevention to treatment interventions. However, there are also other factors that make pediatrics a challenging health topic. As expressed by Albert Ferro, “children are not small adults”^2^—results from adults cannot be directly extrapolated for children. The target population of pediatrics, which includes individuals from neonates to young adults, is also very heterogeneous. The psychological and physical development of children and its interaction with any health condition and intervention need to be considered. Decision‐making and ethical aspects also bring challenges.^3^

A medical study in children or adolescents is not only a study in pediatrics but it is also a study in its own specialty field. Pediatric research therefore benefits from a particularly broad range of available publication platforms—studies in pediatrics are suitable for general medical journals, pediatric journals, and journals specializing on the target health condition. This raises the question as to what extent journals that focus on pediatrics as a whole—a field that is essentially as broad as the entire medicine or even broader—are needed, and what their role in the overall ecosystem of academic debate should be. The aim of this review is to review and summarize the current status of pediatric research and identify gaps in the existing research directions and trends. We will first provide a summary of the most important health issues in children and then provide an overview of existing pediatric journals and research and assess how it matches the existing needs. We will, furthermore, discuss about other existing gaps in pediatric research and conclude with some recommendations for future publications.

## 2 WHAT ARE THE MOST SERIOUS HEALTH ISSUES AFFECTING CHILDREN AND ADOLESCENTS?

**FIGURE 2:**
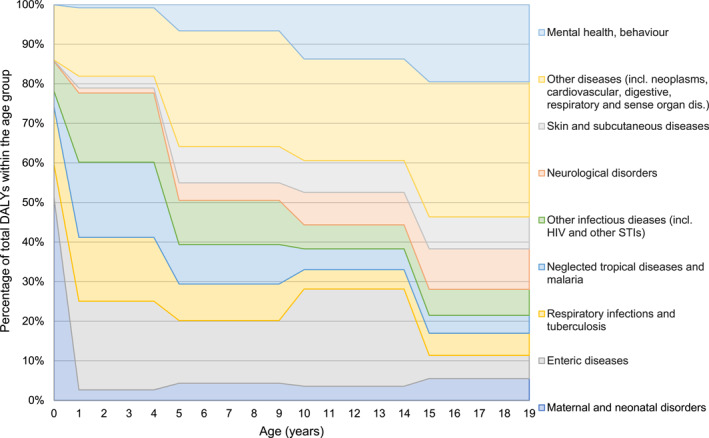
Distribution of disability‐adjusted life years among children and adolescents by cause in 2019. The graph shows the relative contribution of each cause to the total DALYs.

To better understand the research landscape in the context of pediatrics, it is necessary to first review what are the most critical health problems affecting children. The quantification of the impact of diseases is obviously a subjective matter at least to some extent—especially when considering children. However, the Global Burden of Disease study with its worldwide coverage and systematic methodology can provide some insights.^4^ Neonatal conditions and infectious diseases—particularly enteric infections, respiratory infections, and tropical diseases such as malaria—caused about two thirds of the disability‐adjusted life years (DALYs) in children aged <5 years in 2019 (Figure 2).^5^ The total amount of DALYs lost caused by conditions happening in the first year of life are about 10 times higher than that in the subsequent years and 20 times higher than that among children of school age and above. The contribution of infectious diseases decreases over age, whereas noncommunicable diseases (NCDs) and mental health conditions become more common and serious. The two most common groups of NCDs in children and adolescents are skin and subcutaneous diseases and neurological disorders, both contributing to about 7% of all DALYs in school‐aged children and youth. The impact of mental health issues also increases gradually over age, and despite the low number of directly associated deaths, it contributes to about 15% of all DALYs in 15–19 year olds. The growing proportion of injury‐ and violence‐related loss of health is also strongly linked with mental health conditions.^6^

## 3 OVERVIEW OF CURRENT TRENDS IN PEDIATRIC RESEARCH AND PEDIATRIC JOURNALS

A search of PubMed (www.pubmed.gov) with the terms “child”, “adolescent”, “neonate”, “infant”, and “pediatrics” on 1 April 2023 revealed over 4.9 million articles—this comprises about 14% of all records on PubMed. A review of 100 most recent articles showed that the majority (*n* = 68) was published in journals focusing on the clinical topic not restricted to pediatric research. Twenty‐one articles were published in general medical journals. Nine articles were published in specialized pediatric journals and only two in general pediatric journals.

**FIGURE 3:**
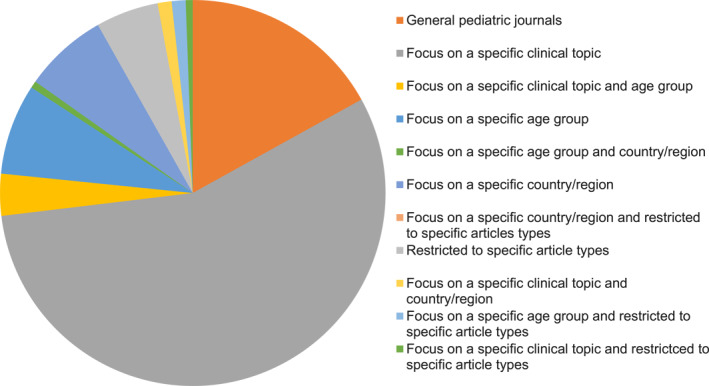
Distribution of journals listed under the category “Pediatrics” in Web of Science (*n* = 171) according to their scope and target. The clinical topic includes journals in subfields of pediatrics and journals on other fields focusing on children and/or adolescents.

General broad‐scope journals were in minority also among the pediatric journals. As of February 2023, the Web of Science lists 171 English language journals in the category of Pediatrics.^7^ We divided the journals according to their main topic, with the aim to separate journals with a broad scope from journals specializing on subtopic of pediatrics, certain age group, study or article type, or geographic location. The distinction was based on a rapid screening of the journal titles, and, if necessary, on the aims and scope of the journal. Twenty‐nine journals did not restrict their topic according to any of the factors mentioned above (Figure 3). The clear majority (*n* = 105), however, specialized in a particular clinical field. Two most common fields were neurology (15 journals) and mental health (eight journals; Table 1). Five journals focused on infectious diseases and two journals on dermatology. A total of 18 journals focused on neonatal health (some also covering the health of pregnant women) and four journals on infants but only two journals on adolescent health.

**TABLE 1:** Distribution of clinical topics of journals restricted to a subfield of pediatrics (*N* = 105).

Clinical topic	*n* (%)
Neurology	15 (14.3%)
Psychiatry and mental health	8 (7.6%)
Breastfeeding and nutrition	7 (6.7%)
Surgery	6 (5.7%)
Infectious diseases and tropical medicine	5 (4.8%)
Orthopedics, exercise medicine, and physical therapy	5 (4.8%)
Dentistry	4 (3.8%)
Hematology and oncology	4 (3.8%)
Health care	3 (2.9%)
Cardiology	3 (2.9%)
Emergency medicine and critical care	3 (2.9%)
Endocrinology	3 (2.9%)
Gastroenterology and nutrition	3 (2.9%)

*Note*: Fields with at least three pediatric journals are shown.

Next, we selected the two general pediatric journals with clearly highest impact factors (JAMA Pediatrics and Lancet Child and Adolescent Health) and reviewed the titles of the original and reviewed articles of the five last available issues, as well as three older issues (those released closest to mid‐2019, mid‐2020, and mid‐2021), to get an overview of time trends and impact of the COVID‐19 pandemic. We also reviewed two relatively new general pediatric journals—Global Pediatric Health and Pediatric Investigation, both established in 2017—and retrieved the topics of original articles published in the past six months (since September 2022). The 113 articles from JAMA Pediatrics and Lancet Child and Adolescent Health covered a broad range of health topics and study designs. As can be expected due to their high impact factor and the established status of their parent journals, randomized controlled trials as well as large‐scale systematic reviews and cohort studies were common. While the majority of articles focused on a clinical question, about one fourth (*n* = 32, 28.3%) of the articles had an epidemiological or societal perspective. Fifteen articles were on neurological disorders, most of which were about autism. Despite us including two issues published in the middle of the COVID‐19 pandemic, only 13 COVID‐19 related articles were included, and most of them are in the recent issues of JAMA Pediatrics. Mental health was the research topic in six articles, all published in JAMA Pediatrics.

The two newer journals had a slightly narrower scope. Global Pediatric Health (*n* = 43 articles) published relatively many case reports on specific, often rare, diseases or conditions; 12 articles were epidemiological rather than clinical, which was in line with the highest‐impact journals. Pediatric investigation, on the other hand, had a much lower number of articles (*n* = 12). We identified only two purely methodological articles in reviewed issues of all four journals. One was a systematic review to identify core outcome sets for child health published in JAMA Pediatrics^8^ and one about the metrics to be used for malnutrition in children affected by HIV published in Global Pediatric Health.^9^

Finally, we also reviewed the titles of the 100 most recent preprint articles from the medRxiv repository. Neurological disorders (*n* = 13 articles) were also common among the preprints. Eleven articles were related to COVID‐19, its complications, or the pandemic. In addition to 34 articles on neonatal health, three articles considered pregnancy and delivery. Nineteen articles presented different tools or methods for evaluation, prediction, or knowledge translation. Two articles on research methodology, one about Bayesian methods for adapting trial data from adults to pediatric populations^10^ and one about the violations of requirements in clinical trials,^11^ were also among the latest preprints.

## 4 WHAT ARE THE MAIN GAPS IN PEDIATRIC RESEARCH, AND WHAT SHOULD PEDIATRIC JOURNALS PAY ATTENTION TO?

Pediatric research is still being primarily disseminated through nonpediatric journals, mainly in journals focusing on specific clinical topics. Most pediatric journals also focus on a specific clinical field. The top health concerns of the pediatric population—neonatal disorders, neurological disorders, mental health of adolescents, and infectious diseases—tend to be well represented in all steps of the publication cascade, including preprints, pediatric journals' focus areas, and published pediatric articles. We, however, also identified some gaps: for example, studies in dermatology were relatively rare, and we also found very few purely methodological studies.

The choice of the target journal is often influenced by a number of factors. General medical journals—including most well‐known and highest‐impact journals as well as an increasing number of “open access mega journals”,^12^—obviously have their advantages. Specialists in different clinical fields may set preference to the journals in their own field that they are more familiar with or because of the fact that results from children are also relevant to the entire field. However, this also means that platforms for scientific debate that take the perspective of children and adolescents are underutilized.

Adequate, transparent, and rigorous methodology is the fundament of research. We argue that just as research resulting from adults are not universally generalizable for children, the special pediatric context should also be taken into account in research methods. As an example, children have very different contact patterns than the adult population, and the assumption of homogeneous mixing commonly used in representations of infectious disease transmission for the general population can lead to very biased results if applied to children.^13^ The role of age as a cofactor is also very different from adults, as a difference of few years in age can already mean a major difference in the child's physiological and psychological characteristics. Nevertheless, published studies on research methods developed or adapted specifically for children were very rare. We could not find any pediatric journal specializing in research methodologies nor did any of the journals we reviewed have a distinct section for methodological articles. In contrast, several methodological articles—including both statistical, predictive, and other techniques for clinical practice as well as articles on research methods–were found among the preprints we screened. Thus, it is apparent that methodological research is being conducted, but there may be obstacles on the way toward publication.

We also identified other gaps. Skin and subcutaneous diseases cause about 7% of all DALYs among children aged from 5 to 14 years; nevertheless, this disease area was underrepresented among the snapshot of articles and journals we reviewed. A reason could be related to the almost minimal mortality associated with skin diseases, which in turn can lead to an underestimation of the value of research in this field. Dermatological diseases can, however, also have psychological consequences which up to now have been largely neglected.^14^ Mental health has also a similar situation—contributing substantially to DALYs but only minimally to mortality. It was promising to see that the importance of mental health has been acknowledged by the journals.

The term “child” can be understood in different ways, whereas in the broad sense, a “child” can mean any minor person; it is also a common term used for children of preschool and primary school age specifically—older than infants but younger than adolescents.^15^ This age group has also a unique disease and morbidity profile, but because of the terminology, it is challenging to identify journals and articles that address particularly this age group. This is one example of gaps that exist for reporting research. Terms such as “infants”, “adolescents”, and “youth” have formal definitions proposed, for example, by the World Health Organization (WHO), which may not always coincide with the colloquial use of these terms.^16^ Moreover, in 2021, Diaz and colleagues called for a standardization of age disaggregation in data analyses, recommending the use of 5‐year strata for all age groups except for those under 5 years.^17^ This is an important step toward standardization, and the 5‐year bins coincide well with the WHO definitions. However, taking into account the rapid development of children which does not always follow the age linearly, a strict stratification of 5‐year age groups may not be the best option in all situations. Therefore, there is an urgent need for more guidance on how to stratify, analyze, and report data from children.

There exists a great number of reporting guidelines for almost any study type, and many of these guidelines have been further extended for specific study types.^18^ However, specific reporting guidelines for pediatric research are still lacking. There exists an extension of STROBE for neonatal infections,^19^ but to our knowledge none of the checklists listed on EQUATOR have an extension that would apply to pediatric research in general. The extensions of RIGHT, CONSORT, PRISMA, and SPIRIT for children are under development^20^, ^21^; it is worth noting that a draft list of items for CONSORT was proposed already in 2010 but not endorsed.^22^ The standardization of reporting, therefore, needs improvement. Characteristics unique for children, such as the balance between the rights for independent decision‐making and parental authority, cannot be covered by checklists intended for the general population. We therefore want to bring into discussion an idea whether a “meta‐checklist”—a guidance how to address issues specific to research in children and adolescents in any type of publication—would be needed.

This overview has several limitations. We did not use a systematic approach as the objective was only to get an overview of the existing status of pediatric research—both on the level of journals and individual articles. We ranked the journals using the impact factor, the validity of which has been questioned. We focused on the most recent publications only. Selections of only up to 100 records from thousands of articles published every year is prone to high uncertainty, and the recent COVID‐19 pandemic has also caused instability in regular research and publication trends. Nevertheless, we believe our study can assist our journal and others in finding the directions that support a better and more balanced dissemination of research that meets the needs of children.

## 5 CONCLUDING REMARKS AND DIRECTIONS FOR A NEW PEDIATRIC RESEARCH JOURNAL

The world has just recovered from a global pandemic which has resulted in an unprecedented loss of health, life, and resources. The impact of the pandemic on medical research has been somewhat controversial: we have witnessed at the same time a huge influx of COVID‐19‐related publications; on the other hand, research in other topics has suffered from pandemic‐related restrictions.^23^ The pandemic has also modified the ways of disclosing research findings: when evidence was needed urgently, preprint platforms and even social media took a greater role in the dissemination of results. Public funding agencies have made it mandatory to publish the results immediately as preprints^24^; on the other hand, the vast influx of information can make it challenging for clinicians, policymakers, and especially the public to judge the reliability of research findings. This was demonstrated, for example, by a debate in Switzerland on the role of children in the epidemic: the Swiss Society of Pediatricians published a statement essentially calling to refrain from any mitigation measures, which was strongly criticized by the research community.^25^ As the world is now returning back to normal, it is important that journals also learn from, and adapt to, the new situation: take advantage of the new dissemination methods, but also assure that clinical practice and public health decision‐making are based only on rigorously peer‐reviewed evidence.

Based on our review, we can make several recommendations on the directions and role that the new journal Pediatric Discovery should take. First, the focus should be on children and adolescents as a whole. Pediatrics consists of a huge and broad number of subspecialties, but the child should be always in the center. As a general pediatric journal, Pediatric Discovery also has the advantage that it can promote the integration of different research fields from the child's perspective. We need to promote a more holistic approach where diseases in children and adolescents are not seen only as diseases but issues that affect—and are affected by—the entire life of a child. Prioritization of diseases also needs consideration. Children are at the beginning of their life and, therefore, have most to lose—indicators with a lifelong span such as DALYs give a good idea of the magnitude of the total consequences attributable to different conditions.

We also need to actively drive the standardization of terms and methods and a more transparent reporting. As a new journal, Pediatric Discovery has an excellent opportunity to lead the way. Requirement to adhere to rigorous reporting methodology is obviously a must, but the fact that pediatrics still lacks comprehensive guidance means we should go one step further: develop and promote reporting guidance and research methodologies that can become a standard for the entire field.

## AUTHOR CONTRIBUTIONS

*Conceptualization*: Janne Estill and Yaolong Chen. *Performing the literature review and analysis*: Janne Estill. *Interpretation of the findings*: Janne Estill and Yaolong Chen. *Writing first draft*: Janne Estill. *Revision of the draft manuscript*: Janne Estill and Yaolong Chen.

## CONFLICT OF INTEREST STATEMENT

Both authors serve as editors of *Pediatric Discovery*. To minimize bias, they were excluded from all editorial decision‐mading related to the acceptance of this article for publication. The authors have no other conflicts to declare.

## ETHICS STATEMENT

Not applicable.

## Data Availability

Data sharing is not applicable to this article as no new data were created or analyzed in this study.
